# Ancestry effects on type 2 diabetes genetic risk inference in Hispanic/Latino populations

**DOI:** 10.1186/s12881-020-01068-0

**Published:** 2020-06-25

**Authors:** Aroon T. Chande, Lavanya Rishishwar, Andrew B. Conley, Augusto Valderrama-Aguirre, Miguel A. Medina-Rivas, I. King Jordan

**Affiliations:** 1 School of Biological Sciences, Georgia Institute of Technology, 950 Atlantic Drive, Atlanta, GA 30332 USA; 2 IHRC-Georgia Tech Applied Bioinformatics Laboratory, Atlanta, GA USA; 3 PanAmerican Bioinformatics Institute, Cali, Valle del Cauca Colombia; 4 Biomedical Research Institute (COL0082529), Cali, Colombia; 5 Universidad Santiago de Cali, Cali, Colombia; 6 Centro de Investigación en Biodiversidad y Hábitat, Universidad Tecnológica del Chocó, Quibdó, Chocó Colombia

**Keywords:** Polygenic risk score (PRS), Genetic risk, Type 2 diabetes (T2D), Genetic ancestry, Population genetics, Hispanic/Latino (HL), Colombia, Chocó, Antioquia

## Abstract

**Background:**

Hispanic/Latino (HL) populations bear a disproportionately high burden of type 2 diabetes (T2D). The ability to predict T2D genetic risk using polygenic risk scores (PRS) offers great promise for improved screening and prevention. However, there are a number of complications related to the accurate inference of genetic risk across HL populations with distinct ancestry profiles. We investigated how ancestry affects the inference of T2D genetic risk using PRS in diverse HL populations from Colombia and the United States (US). In Colombia, we compared T2D genetic risk for the Mestizo population of Antioquia to the Afro-Colombian population of Chocó, and in the US, we compared European-American versus Mexican-American populations.

**Methods:**

Whole genome sequences and genotypes from the 1000 Genomes Project and the ChocoGen Research Project were used for genetic ancestry inference and for T2D polygenic risk score (PRS) calculation. Continental ancestry fractions for HL genomes were inferred via comparison with African, European, and Native American reference genomes, and PRS were calculated using T2D risk variants taken from multiple genome-wide association studies (GWAS) conducted on cohorts with diverse ancestries. A correction for ancestry bias in T2D risk inference based on the frequencies of ancestral versus derived alleles was developed and applied to PRS calculations in the HL populations studied here.

**Results:**

T2D genetic risk in Colombian and US HL populations is positively correlated with African and Native American ancestry and negatively correlated with European ancestry. The Afro-Colombian population of Chocó has higher predicted T2D risk than Antioquia, and the Mexican-American population has higher predicted risk than the European-American population. The inferred relative risk of T2D is robust to differences in the ancestry of the GWAS cohorts used for variant discovery. For trans-ethnic GWAS, population-specific variants and variants with same direction effects across populations yield consistent results. Nevertheless, the control for bias in T2D risk prediction confirms that explicit consideration of genetic ancestry can yield more reliable cross-population genetic risk inferences.

**Conclusions:**

T2D associations that replicate across populations provide for more reliable risk inference, and modeling population-specific frequencies of ancestral and derived risk alleles can help control for biases in PRS estimation.

## Background

Diabetes mellitus is a global pandemic [1–3]. The prevalence of adult onset (type 2) diabetes has nearly doubled over the last 30 years, and the number of cases has increased by more than 300 million. This increase has been driven largely by modernization and the accompanying changes in diet and lifestyle. According to the International Diabetes Federation (IDF) Atlas [4], 425 million adults worldwide are currently living with diabetes, with half of them remaining undiagnosed. In the United States (US) alone, more than 100 million adults have either prediabetes or diabetes. US Hispanic/Latino (HL) populations bear a disproportionate burden of type 2 diabetes (T2D), with a prevalence almost twice as high as that of non-Hispanic whites [5, 6]. Globally, countries from the Latin America and Caribbean region show the highest diabetes prevalence compared to six other regions.

T2D is a multifactorial disease with a complex set of interacting environmental and genetic causes contributing to its etiology. Historically, risk management for T2D has been focused squarely on environmental factors, with an emphasis on changes in diet and lifestyle. Physicians have been taught to evaluate a suite of clinically measurable risk factors, e.g. weight and blood pressure along with blood sugar and cholesterol levels, in assessing patients’ likelihood of developing T2D. In addition to these clinical features, family history and race/ethnicity are also widely recognized as T2D risk factors, underscoring genetic contributions to disease expression. Indeed, genetic factors have been estimated to account for 20–80% of the variance in T2D development [7–9]. It follows that an understanding of individual patients’ genetic risk should become part of the standard of care for T2D screening and prevention.

Individuals’ risk for common heritable diseases, such as T2D, can be quantified as polygenic risk scores (PRS) [10]. The ability to calculate PRS rests on genome-wide association studies (GWAS), which characterize specific genetic variants (alleles) that increase disease risk [11]. GWAS typically uncover numerous variants across the genome, each of which contributes a small fraction of the overall disease risk. PRS can be computed by summing the number of risk increasing alleles in individuals’ genomes, and scores can be weighted by the effect sizes of the risk alleles [12]. This approach to inferring genetic risk works very well when it is applied to patient cohorts from the same populations where the GWAS were conducted. However, the extent to which genetic risk can be accurately calculated across populations with divergent ancestries is a matter of contention [13, 14]. On the one hand, many GWAS are highly replicable, with the same variants often discovered in multiple populations [15, 16]. On the other hand, recent studies have shown that differences in genetic ancestry can lead to mis-estimation of PRS across populations [17–19].

The challenge of accurate PRS estimation across ancestry groups is particularly pressing for HL populations. First, there is a severe bias towards European ancestry cohorts in GWAS. As of 2006, only 0.06% of GWAS samples were from HL cohorts, and the fraction had only risen slightly to 0.54% by 2016 [20, 21]. Second, HL is a politically inspired, pan-ethnic label that does not correspond to any natural (i.e. genetic) classification of human populations [22]. Individuals with origins in Latin America typically have three-way ancestry contributions from African, European, and Native American source populations, and they can differ dramatically with respect to the relative proportions of each [23–27]. Even neighboring populations from within the same Latin American country can show widely divergent ancestry profiles [28]. Accordingly, the extent to which existing GWAS variants can be used to accurately infer genetic risk among diverse HL populations is currently unknown.

In this study, we explored the relationship between ancestry and T2D genetic risk inference in HL populations from Colombia and the US. We found that T2D genetic risk is positively correlated with African and Native American ancestry and negatively correlated with European ancestry, consistent with epidemiological results. We also show that T2D genetic risk inference holds up well across different GWAS ancestry cohorts and propose an approach whereby ancestry information can be used to support cross-population risk inference.

## Methods

### Diabetes epidemiological data

Data on the worldwide prevalence of diabetes mellitus were taken from The World Bank [29]. Worldwide diabetes prevalence values are expressed as the percentage of the population between the ages of 20 and 79 diagnosed with diabetes. Prevalence values are reported for 264 countries, which were broken down into seven World Health Organization (WHO) regions and four WHO income groups. Data on the prevalence of diabetes for the United States (US) were taken from the American Diabetes Association [30]. US diabetes prevalence values are expressed as the age-adjusted percentage of the population diagnosed with diabetes. Prevalence values are broken down by the US census self-identified race/ethnicity groups and further sub-divided into country/region of origin for individuals who self-identify as Hispanic/Latino (HL). Diabetes prevalence values for the European-American (EA) and Mexican-American (MA) populations were taken from the Utah Department of Public Health [31] and the County of Los Angeles Public Health agency [32]. Note that these US diabetes prevalence values correspond to the specific populations sampled as part of the 1000 Genomes Project and used for genetic risk inference (see Methods section on Type 2 diabetes (T2D) genetic risk inference).

### Genome wide association study (GWAS) data

GWAS data were taken from the NHGRI-EBI GWAS Catalog [11]. All reported GWAS (as of 3/31/2018) were characterized with respect to the trait under consideration and the ancestry of the study cohort. GWAS cohorts were characterized as African, East Asian, European, Hispanic/Latino, or Native American following the GWAS Catalog framework for representation of ancestry data in genomic studies [33]. The total number of single-nucleotide polymorphism (SNP) associations that reach the GWAS Catalog significance threshold (*P* < 1 × 10^− 5^) were recorded for each GWAS trait. For each T2D SNP association, we recorded the study (ies) where it was reported, the cohort ancestry, the SNP identifier, its chromosomal location, and the identity of the trait-increasing effect allele. T2D GWAS summary statistics for a trans-ethnic meta-analysis, which integrated cohorts with four distinct ancestries, were taken from the DIAGRAM consortium [34, 35]. For these data, the GWAS SNP effect alleles, ancestry-specific directions of effect, effect sizes, and *P*-values were recorded.

### Type 2 diabetes (T2D) genetic risk inference

Whole genome sequences from the 1000 Genomes Project [36] and imputed whole genome genotypes from the ChocoGen Research Project https://www.chocogen.com [37] were used for T2D polygenic risk score (PRS) calculation (Table 1). For the 1000 Genomes Project data, SNP data were taken from the phase 3 data release for one Colombian population – Colombians from Medellín, Colombia – and two US populations: Utah Residents (CEPH) with Northern and Western European Ancestry and Mexican Ancestry from Los Angeles USA. The 1000 Genomes Project human genome sequence data are de-identified and made publicly available for research use without restriction. For the ChocoGen Research Project, whole genome genotypes for sample donors were characterized using the Illumina HumanOmniExpress-24 SNP array as previously described, yielding ~ 500,000 SNPs per individual [28, 37]. The genotypes were imputed using the program IMPUTE2 [38] with the 1000 Genomes Project phase 3 haplotype reference panel [39] as previously described [40], yielding ~ 35 million additional SNPs across all samples. The ChocoGen project was conducted with the approval of the Ethics Committee of the Univerisidad Tecnológica del Chocó (ACTA N^o^ 01-v1), and all sample donors signed informed consent documents.

**Table 1:** Populations analyzed in this study

Data Source^**a**^	Population Description	Population Name	***n***^***b***^
ChocoGen	Chocoano in Quibdó, Colombia	Chocó	94
1KGP	Colombian in Medellin, Colombia	Antioquia	94
1KGP	Yoruba in Ibadan, Nigeria	African	108
1KGP	Iberian populations in Spain	European	107
1KGP	Utah residents with NW European ancestry	European-American (EA)	99
1KGP	Mexican Ancestry from Los Angeles USA	Mexican-American (MA)	64
1KGP	Peruvian in Lima, Peru	Native American	85

^a^1KGP = 1000 Genomes Project

^b^*n* = number of sample donors per population

For each individual genome, an unweighted T2D PRS was computed by calculating the normalized sum of the number of T2D SNP effect alleles found in the genome [12]. It should be noted that T2D PRS were not weighted by SNP effect sizes owing to the fact that the T2D SNP associations used here were curated from multiple studies whose effect sizes cannot be accurately combined [16]. T2D PRS were calculated as:

$$ PRS={\sum}_{i=1}^n{G}_i/{\sum}_{i=1}^n{A}_i $$

where *G*_*i*_ ∈ {0, 1, 2} corresponds to homozygous absent, heterozygous, and homozygous present effect alleles for each T2D SNP *i* and *A*_*i*_ ∈ {0, 1, 2} corresponding the total number of alleles with variant calls at each SNP *i*. T2D PRS were compared to individuals’ continental genetic ancestry fractions – African, European, and Native American – which were taken from our previous studies [28, 40].

T2D PRS were computed for the Colombian and US populations using an unpruned set of 165 T2D-associated SNPs along with a reduced linkage disequilibrium (LD) pruned set of 42 SNPs (Additional file 1: Table S1). LD pruning was performed on the four Colombian and US populations analyzed here using the program PLINK [41] with 2000 SNP window size and a threshold of *r*^*2*^ > 0.1, where *r*^*2*^ corresponds to the level of linkage disequilibrium between pairs of SNPs in the window. An additional round of LD clumping was performed on the DIAGRAM GWAS summary statistic data using the LDpred program, with the same suggested window size of 2000 SNPs [42]. LDpred uses the LDscore method to choose the highest effect size SNP for each LD window and subsequently reweights the effect sizes for all retained SNPs.

### Genetic ancestry and T2D risk

The program ADMIXTURE was used to compute the three way continental ancestry percentages – African, European, and Native American – for all individuals from the Colombian and US populations analyzed here [43]. The modern Colombian and US populations were compared to the proxy ancestral reference populations shown in Table 1, with ADMIXTURE run for K = 3 ancestral components, corresponding to each of the three continental population groups that admixed to form modern American populations. This process yields a vector of three ancestry fractions for any individual admixed genome sampled from the modern populations: f_African_, f_European_, f_NativeAmerican_ (Additional file 2: Figure S1). Then, for each of the three continental ancestry components, individuals’ continental ancestry fractions were regressed against their T2D PRS using unweighted ordinary least squares regression (OLS) with the lm function in R:

$$ {PRS}_i=\alpha +\upbeta {x}_i+{\varepsilon}_i $$

where *PRS*_*i*_ is the predicted polygenic risk score for individual *i*; α and β are constants describing the intercept and slope, respectively; *x*_*i*_ is the ancestry fraction for individual *i*; and *ε*_*i*_ is an error term describing the deviation from the fitted line. The resulting OLS produces: β_0_, the model β or slope; the standard error of the model; the *r*^2^ value describing the model’s fit; the model t-statistic; and a two-tailed *P*-value. For visualization purposes, a best fit line with confidence intervals was computed using local polynomial regression (loess).

## Results

### Diabetes prevalence and population disparities

**Fig. 1:**
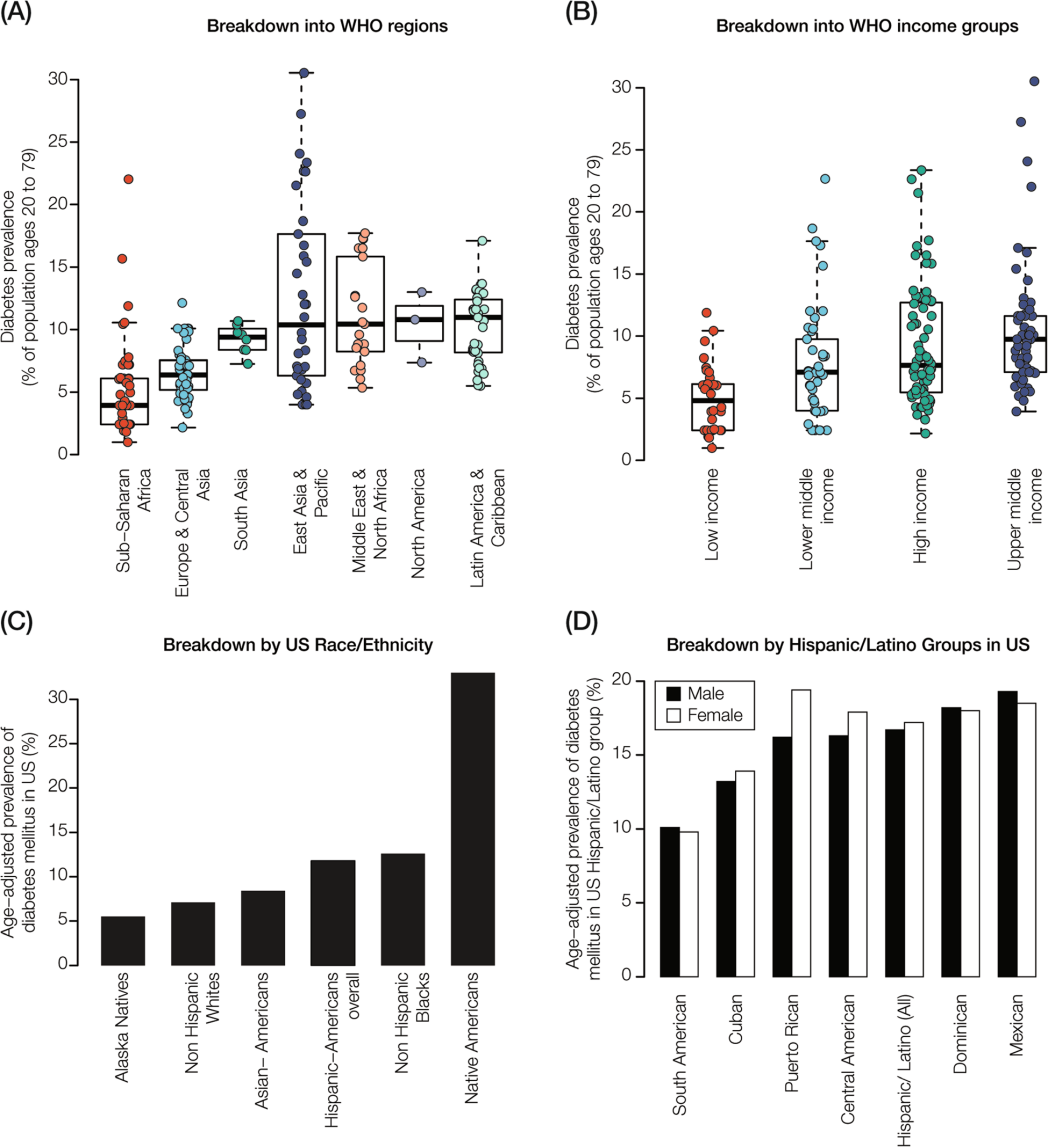
Diabetes global prevalence and population disparities. **a** Diabetes prevalence distributions shown for (**a**) the seven world health organization (WHO) geographic regions and (**b**) the four WHO income groups. (**c**) Diabetes prevalence for United States (US) census race/ethnicity groups. **d** Hispanic/Latino (HL) diabetes prevalence in the US broken down by country (region) of origin and shown separately for males (black) and females (white)

Diabetes is characterized by an extremely high disease burden along with pronounced disparities in prevalence among countries, regions, and income groups worldwide (Fig. 1a and b). It should be noted that, while these prevalence data are not broken down into diabetes types, the vast majority of diabetes cases correspond to adult onset, non-insulin dependent, type 2 diabetes (T2D). The US is no exception to this trend; there is a high overall diabetes prevalence in the country and marked disparities among racial and ethnic groups (Fig. 1c). Native Americans, African Americans, and Hispanic/Latino (HL) populations bear a disproportionately high share of the diabetes disease burden in the US compared to Asian Americans and European Americans. Interestingly, there are also notable disparities within ethnic groups. HL populations with distinct origins in Latin America can have very different diabetes prevalence (Fig. 1d). Individuals from South America show diabetes prevalence close to what is seen for Asian Americans, whereas Mexican Americans show a two-times greater prevalence, close to what is seen for Native Americans. Among HL regional groups, diabetes prevalence can also differ between males and females in a group-specific manner.

The observed diabetes prevalence disparities among HL groups with distinct origins begs an explanation. Diabetes is a complex common disease with multifactorial causes, including genetic and environmental effects along with interactions between them. Nevertheless, T2D in particular is strongly genetically influenced with estimates of heritability ranging from 20 to 80% [7–9]. Furthermore, genetic ancestry is known to impact the burden T2D; both African and Native American ancestry have been associated with increased T2D prevalence [44–48]. Thus, one may naively expect to observe more uniformity in T2D prevalence within a single ethnic group. But the pan-ethnic HL label does not in fact correspond to a ‘natural’ group with a shared genetic ancestry. Rather, HL groups encompass an extraordinarily diverse set of populations, which are characterized by distinct combinations of ancestry from Africa, Europe, and the Americas [23–27]. Additionally, the Native American component of HL ancestry varies substantially according to the regional origins of the populations [28, 49, 50]. With this in mind, we have been investigating the contributions of ancestry to genetic risk and T2D health disparities in diverse HL populations.

### GWAS ancestry bias and T2D risk inference

The power to infer genetic risk for complex common diseases, such as T2D, has exploded in recent years owing to the accumulation of GWAS for a wide variety of health-related traits [10, 11]. GWAS yield lists of trait SNP associations, including the identity of trait-increasing effect alleles, each of which slightly increases the risk of disease. Accordingly, an individual’s genetic risk for a given trait can be estimated as a polygenic risk score (PRS), which is calculated as the normalized sum of risk (effect) alleles encoded in their genome. However, the overwhelming bias towards European cohorts in GWAS [20, 21] presents a major challenge to this paradigm. Specifically, the extent to which PRS can be accurately inferred across population groups with distinct ancestry profiles is a matter of great concern [13, 14]. On the one hand, many robust SNP associations are known to replicate across populations [15, 16]. On the other hand, GWAS SNP ascertainment biases and demographic process have been shown to yield systematic errors in PRS calculation across populations [17–19].

**Fig. 2:**
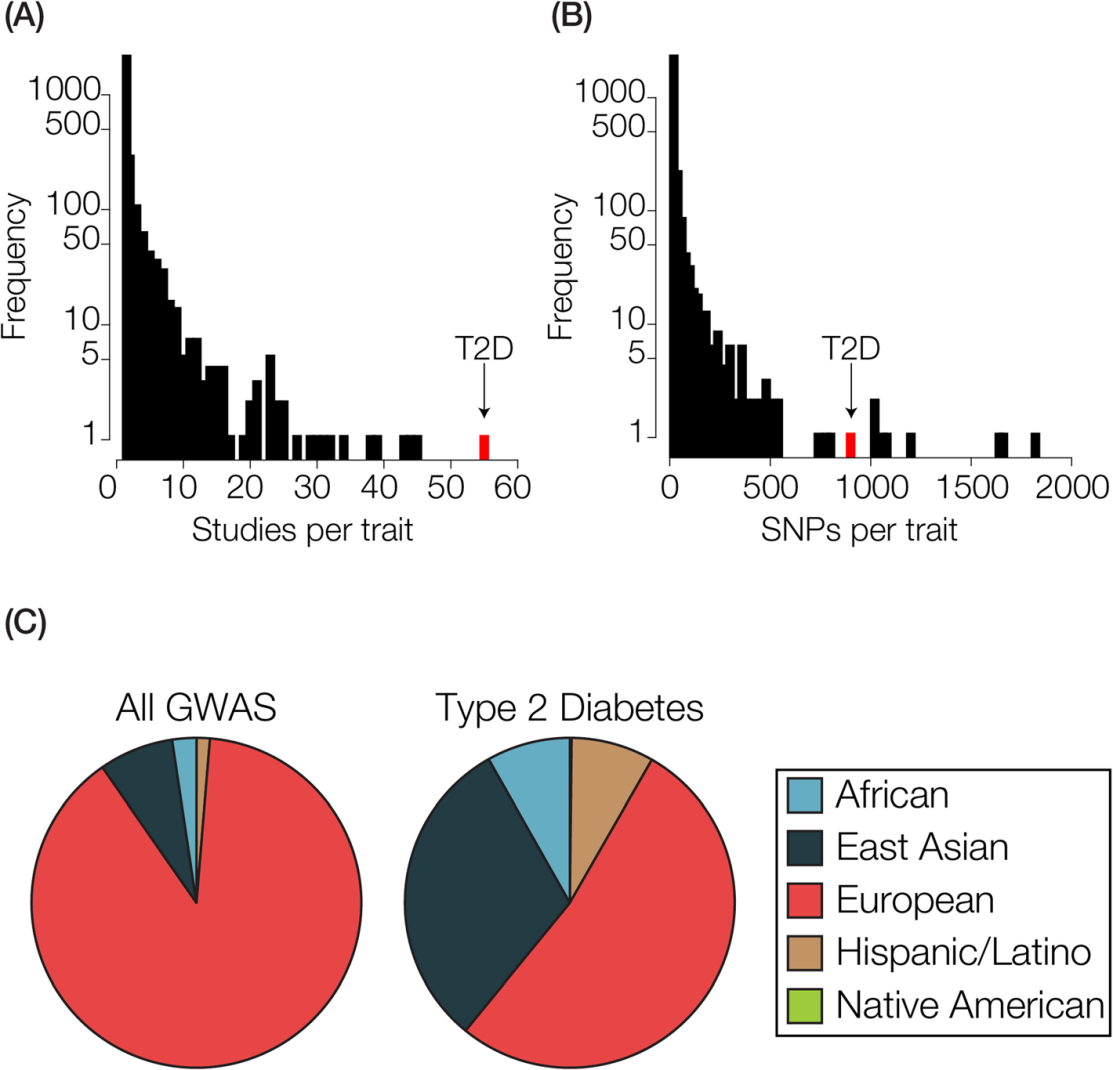
Genome wide association studies (GWAS) on type 2 diabetes (T2D). The number of (**a**) GWAS and the number of (**b**) SNP-associations per GWAS trait are shown, with T2D values in red. **c** The fractions of continental ancestry groups represented in GWAS cohorts are shown for all GWAS and for T2D GWAS alone

Here, we aimed to explore the effects of ancestry on the calculation of PRS for T2D across diverse populations. In support of this effort, we found that T2D is distinct compared to GWAS for most other traits in several respects, largely owing to the intensity of focus on the genetic architecture of the disease and its epidemiological importance for populations across the world. T2D has the most independent studies of any trait in the NHGRI-EBI GWAS catalog (Fig. 2a), and it has among the most SNP associations reported for any trait (Fig. 2b). Perhaps even more importantly, for our purposes, T2D cohorts show substantially more ancestry diversity than typical GWAS traits (Fig. 2c). A slight majority of T2D GWAS cohorts have European ancestry, but there are substantial number of cohorts with East Asian, African, and HL ancestry. A number of T2D GWAS have employed a trans-ethnic study design, whereby cohorts with distinct ancestries are combined in an effort to increase the reliability of discovered SNP associations [34, 35]. Taken together, the large number of T2D studies with diverse ancestry cohorts and the large number of T2D associations provide resolution for our efforts to (i) calculate PRS across diverse populations and (ii) assess the impact of ancestry on predicted T2D genetic risk.

### Ancestry and T2D genetic risk inference: Colombia

**Fig. 3:**
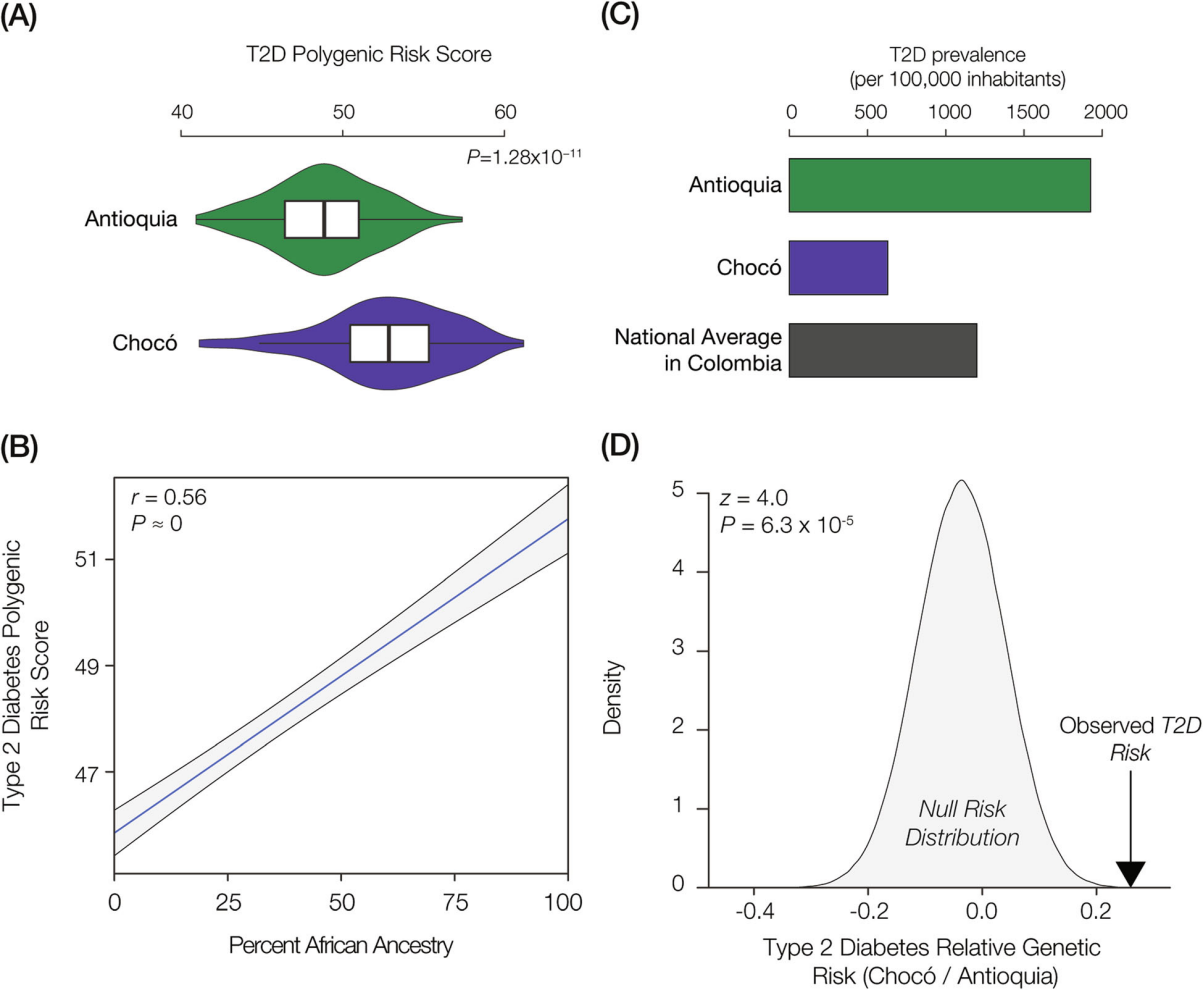
T2D genetic risk and observed prevalence in Colombia. **a** T2D polygenic risk score distributions are shown for Antioquia (green) and Chocó (purple). **b** T2D polygenic risk scores for individuals from Antioquia and Chocó regressed against their percent African ancestry. **c** Observed T2D diabetes prevalence for Antioquia (green), Chocó (purple), and Colombia overall (gray). **d** Observed T2D relative genetic risk Chocó/Antioquia compared to the null distribution of relative genetic risk between the two populations

We first explored the relationship between ancestry and T2D genetic risk for the Colombian populations of Antioquia and Chocó. Despite the fact that these two administrative departments (states) share a common border, their populations were historically isolated and show very distinct ancestry profiles. The population of Antioquia has majority European ancestry (75%) followed by Native American (18%) and African (7%) fractions, whereas the ancestry of Chocó is primarily African (76%) with smaller European (13%) and Native American (11%) components [28]. Genome sequences were characterized for individuals from the two populations and T2D PRS were computed for all individuals as described in the Methods. The distributions of T2D PRS for the two populations were then compared in order to assess their relative genetic risk. Consistent with previous results [40], we found that Chocó has significantly higher predicted genetic risk for T2D compared to Antioquia (Fig. 3a), and the higher genetic risk for T2D in Chocó is correlated with African ancestry (Fig. 3b). The elevated T2D risk for Chocó can be observed when all 165 T2D-associated SNPs are used for PRS calculation (Fig. 3) or when a reduced set of 42 linkage disequilibrium (LD) pruned SNPs is used (Additional file 2: Figure S2 panels A & B). These findings are consistent with reports from the US showing a correlation between T2D genetic risk and African ancestry [51], and African Americans are known to have substantially higher T2D prevalence compared to European Americans [44, 46–48]. In Colombia however, Antioquia shows approximately three-times higher observed T2D prevalence compared to Chocó (Fig. 3c), in direct contrast to the predicted genetic risk for the two populations and the epidemiological data from the US.

We previously attributed the difference between the relative predicted genetic risk of T2D for the two Colombian populations and their observed T2D prevalence to gene-by-environment interactions, whereby diet and lifestyle in Chocó serve as protective factors against T2D [40]. However, another possible explanation for this discrepancy is that there is a systematic bias in T2D PRS calculations across populations of this kind with distinct ancestry profiles [17–19]. We addressed this possibility by comparing the observed T2D relative risk for Chocó / Antioquia to a null distribution of relative risk generated by permuting 500,000 random sets of GWAS SNPs (risk alleles) of the same size as the T2D SNP set. If there were a systematic bias in the population-specific frequencies of GWAS risk alleles for the two populations, then the null distribution would be expected to show an overall increase of genetic risk in Chocó. We do not observe any such bias; the observed relative risk of T2D is significantly greater than the null expectation (Fig. 3d).

**Fig. 4:**
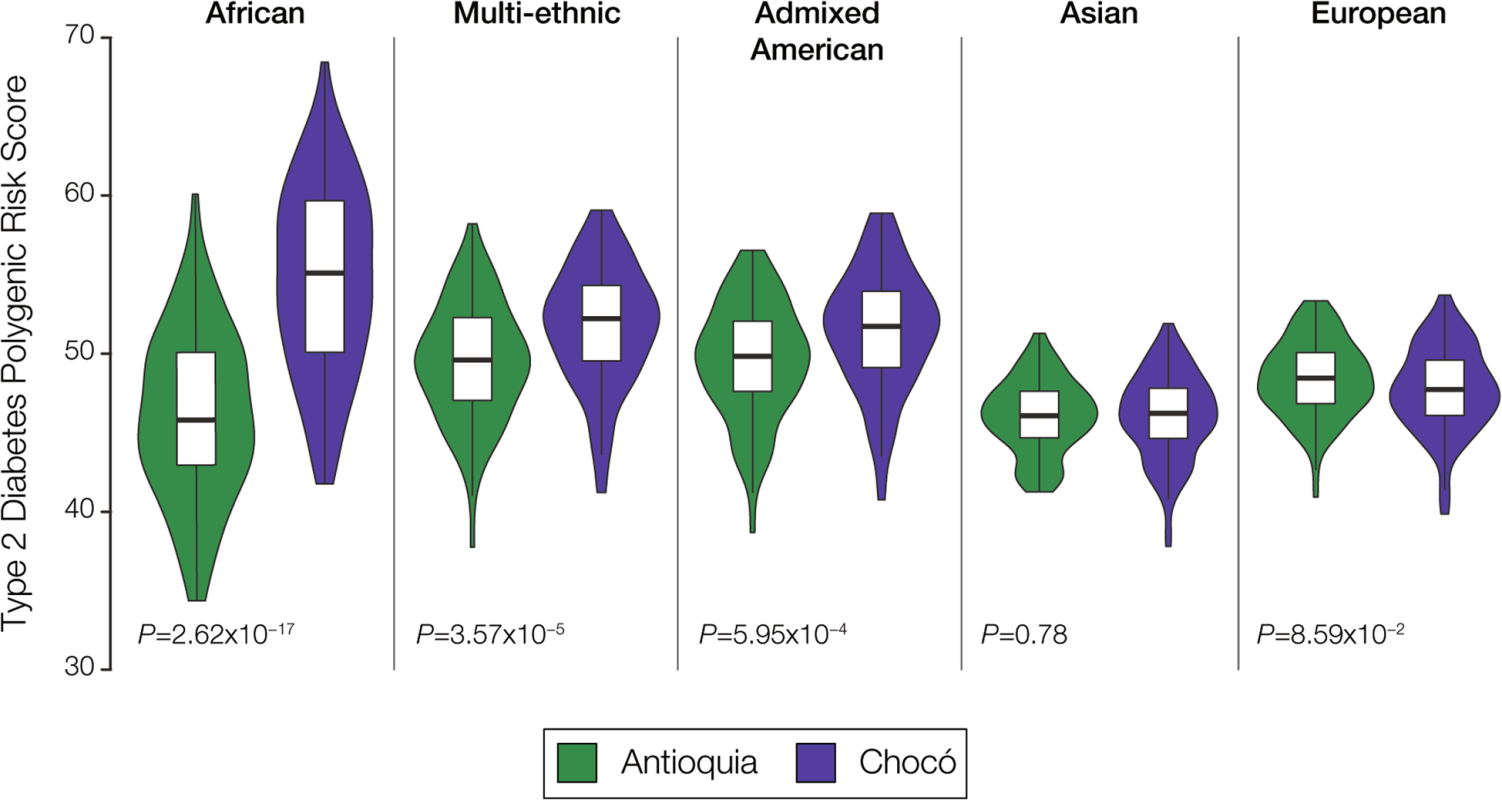
T2D genetic risk comparison in Colombia based on different GWAS cohort continental ancestries. T2D polygenic risk score distributions for Antioquia (green) and Chocó (purple) are shown for SNP associations discovered in patient cohorts with distinct continental ancestries

As previously described, the major source of bias for cross-population PRS calculation is attributed to the vast over-representation of European cohort GWAS. It is possible that GWAS SNPs discovered in European study cohorts will not accurately capture genetic risk in non-European cohorts. This problem could be even more exacerbated in the case of the admixed Colombian populations studied here, one of which looks more European while the other is more African. The fact that T2D has been the subject of numerous GWAS across diverse population cohorts (Fig. 2) provides an opportunity to interrogate this potential bias. To do so, we characterized T2D GWAS variants according to the ancestry of the study cohorts where they were discovered and then re-calculated population-specific T2D PRS distributions for each ancestry separately. We were able to classify T2D SNPs into five different ancestry profiles, three of which showed significantly higher risk in Chocó and two of which yielded no significant difference (Fig. 4). None of the comparisons showed significantly higher T2D risk in Antioquia, and all of the cohorts with ancestry most similar to the Colombian populations (African, Multi-ethnic, and Admixed American) showed higher relative risk in Chocó. These results support the finding of higher genetic risk for T2D in Chocó, associated with African ancestry, and do not suggest that this finding can be attributed to GWAS SNP discovery bias.

### Ancestry and T2D risk inference: United States (US)

**Fig. 5:**
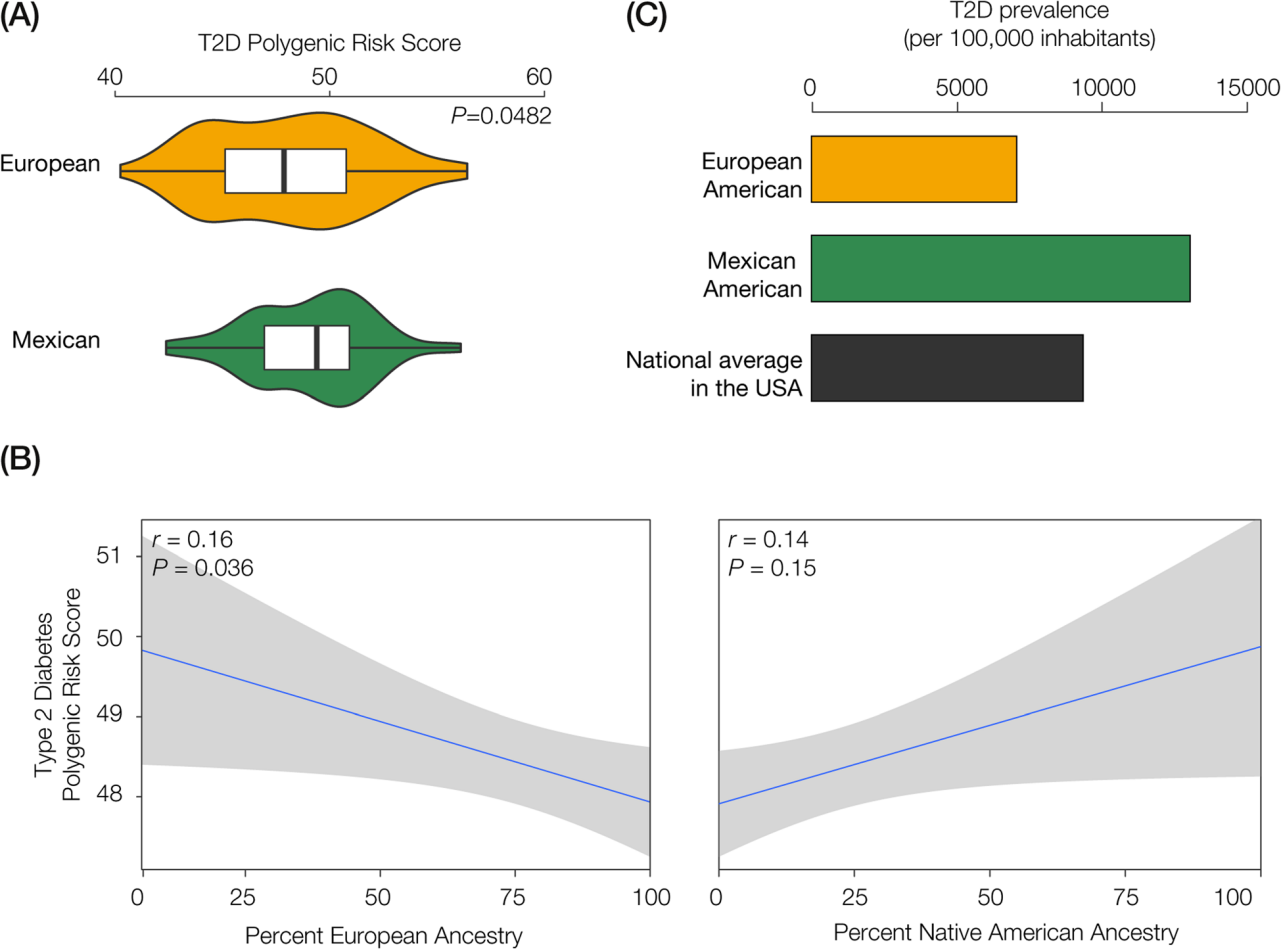
T2D genetic risk and observed prevalence for European-American (EA) and Mexican-American (MA) cohort populations. **a** T2D polygenic risk score distributions are shown for EA (gold) and MA (green). **b** T2D polygenic risk scores for EA and MA individuals are regressed against their percent European and percent Native American ancestry. **c** Observed T2D diabetes prevalence values for EA (gold), MA (green), and the United States overall (gray)

We performed a similar comparison of T2D genetic risk for European-American (EA) and Mexican-American (MA) populations in the US. With the same set of T2D SNPs used to compare genetic risk in Colombia, the MA population shows marginally higher T2D genetic risk than the EA population (Fig. 5a). As was the case for Colombia, the same differences in T2D genetic risk between the US populations can be seen when all 165 T2D-associated SNPs are used for the PRS calculations (Fig. 5a) or when a reduced set of 42 linkage disequilibrium (LD) pruned SNPs is used (Figure S2 panels C & D). For these two US populations, T2D genetic risk is negatively correlated with European ancestry and positively correlated with Native American ancestry (Fig. 5b). However, unlike what we observed in Colombia, the relative genetic risk estimates between the two populations are consistent with the observed T2D prevalence; the MA population shows approximately two-times higher T2D prevalence than the EA population (Fig. 5c).

**Fig. 6:**
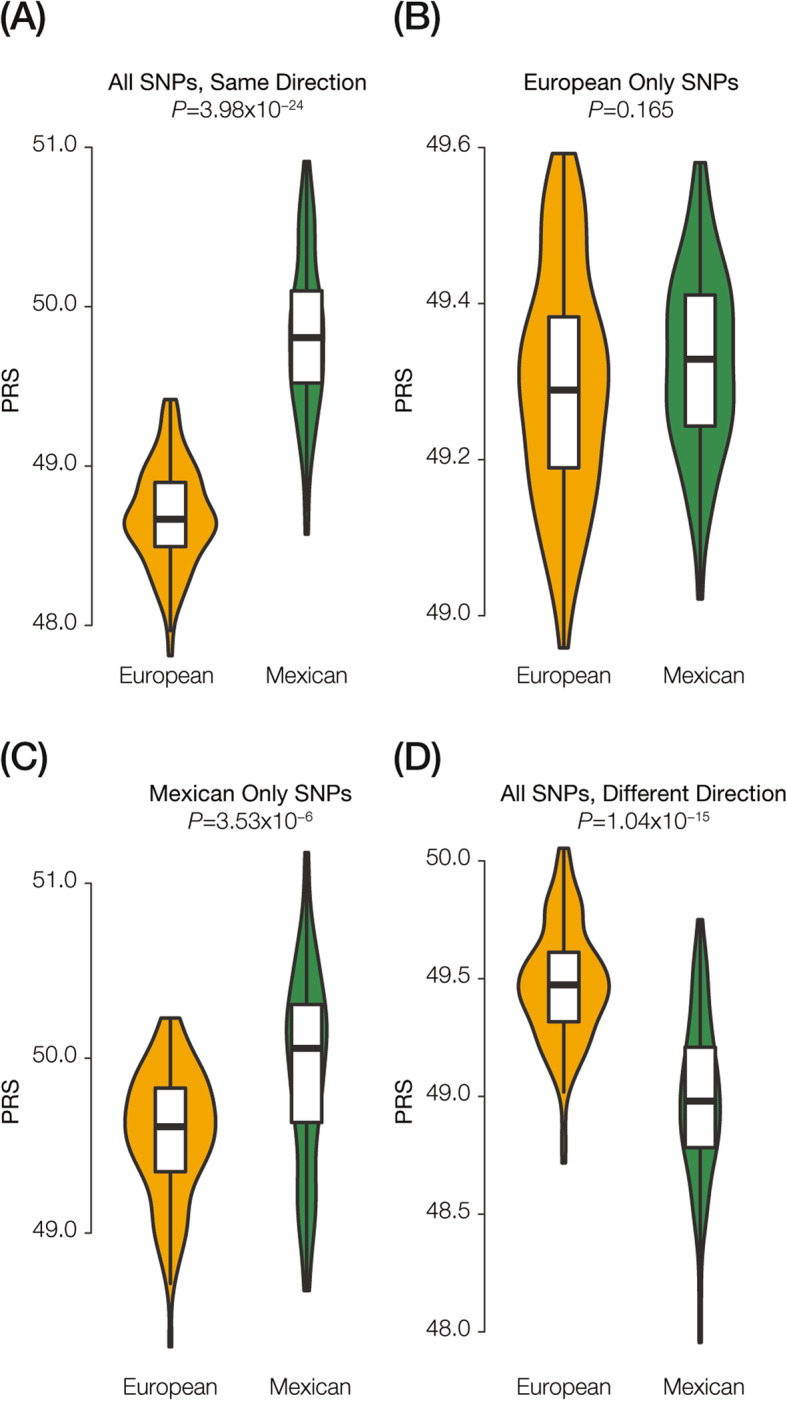
T2D genetic risk comparison between European-American (EA) and Mexican-American (MA) cohort populations based on ancestry-specific SNP effects. T2D polygenic risk score distributions for EA (gold) and MA (green) populations are compared for (**a**) all SNPs with consistent ancestry effects, (**b**) SNPs with European ancestry-specific effects, (**c**) SNPs with Mexican ancestry-specific effects, and (**d**) SNPs with opposing ancestry effects

Despite the consistency of the T2D genetic risk estimates and the observed prevalence values for these two populations, we wanted to further explore the contribution of genetic ancestry differences to potential biases in genetic risk calculation. To do so, we took advantage of a recent trans-ethnic GWAS meta-analysis [34, 35] to curate T2D SNPs that were discovered in one or more cohorts with distinct ancestries, including European and Mexican ancestry cohorts. We then computed T2D PRS distributions using (i) significant SNPs that showed the same direction of effect between the two ancestry cohorts, (ii) SNPs that were significant in the European ancestry cohort only, (iii) SNPs that were significant in the Mexican ancestry cohort only, and (iv) SNPs that showed different directions of ancestry-specific effects (Fig. 6). The SNPs with effects that are shared between populations or effects that are population-specific all yielded higher T2D PRS in the MA compared to the EA population. The magnitude and significance of this relationship were most pronounced for the ancestry shared SNPs (Fig. 6a). The SNPs with different effects between the two ancestry cohorts were the only ones that showed higher T2D PRS in the EA population (Fig. 6d). These results underscore the potential utility of combining cohorts with distinct ancestries for GWAS SNP discovery, in terms of both increasing the reliability of SNP effect allele discovery and decreasing the likelihood of false discoveries. Indeed, we found that the T2D SNPs that showed shared effects across ancestry cohorts had effect size odds-ratio (OR) values almost an order of magnitude higher than SNPs with divergent ancestry-specific effects (Shared OR = 2.40 versus Divergent OR = 0.28).

### Correcting for ancestry bias in T2D risk inference

A number of recent studies have underscored (i) the extreme bias of European ancestry cohorts in GWAS [20, 21] and (ii) the corollary potential to mis-estimate genetic risk across populations with diverse ancestries [13, 14, 17–19]. Kim et al. identified two potential sources of bias for cross population ancestry risk inference [18], which we will call here SNP ascertainment bias and SNP discovery bias. SNP ascertainment bias is related to the fact that SNP microarrays are typically used for genotyping in GWAS, and these microarrays are designed, for the most part, to capture high minor allele frequency (MAF) SNPs in European populations. This will lead to the ascertainment of SNPs with higher MAF in European populations compared to other global populations, particularly populations from Africa that are enriched for ancestral alleles [52]. Then, systematic differences in the proportions of derived alleles, which most often correspond to the minor allele, versus ancestral alleles, may lead to directional biases in the estimation of genetic risk. SNP discovery bias is related to the increased power of GWAS to detect SNPs with higher MAF. Irrespective of microarray design, discovery of SNPs in European cohorts will yield relatively higher MAF in European populations compared to other populations, which can also lead to mis-estimation of genetic risk across populations with distinct ancestries.

Here, we propose a potential control for these two sources of PRS bias, based on correction for systematic differences in the proportions of ancestral versus derived alleles in populations with distinct ancestry profiles. Ancestral alleles tend to correspond to major alleles, whereas derived alleles most often correspond to minor alleles in discovery cohort populations. While GWAS risk alleles can be more evenly distributed across ancestral (44%) versus derived (56%) alleles, differences in the frequencies of these allele classes across populations can still introduce bias in genetic risk inference [18]. The idea behind the control that we propose here is to eliminate any possible bias owing to population-specific differences in the frequencies of ancestral versus derived alleles, which are mainly attributed to demographic factors (i.e. genetic drift).

The steps in the control are shown below. Further detail regarding the execution of each individual step are provided in Additional file 2 (see pages 5–7).

1. Collect trait SNP set and calculate population-specific PRS values and between-population PRS differences (*∆PRS*).
2. Determine the distribution of derived allele frequencies (DAF) for trait-associated SNPs in the GWAS cohort source population.
3. Randomly sample SNP sets parameterized by this DAF distribution based on the DAFs from the distinct populations being compared (thereby eliminating between-population DAF biases).
4. Calculate between-population *∆PRS* for all randomly sampled SNP sets and determine the null *∆PRS* distribution.
5. Compare the observed *∆PRS* to the null *∆PRS* distribution and compute a z-score as the ancestry-corrected *∆PRS*: *corr*. *∆PRS* = (*obs∆PRS* − *μ*_*null∆PRS*_)/*σ*_*null∆PRS*_.

**Fig. 7:**
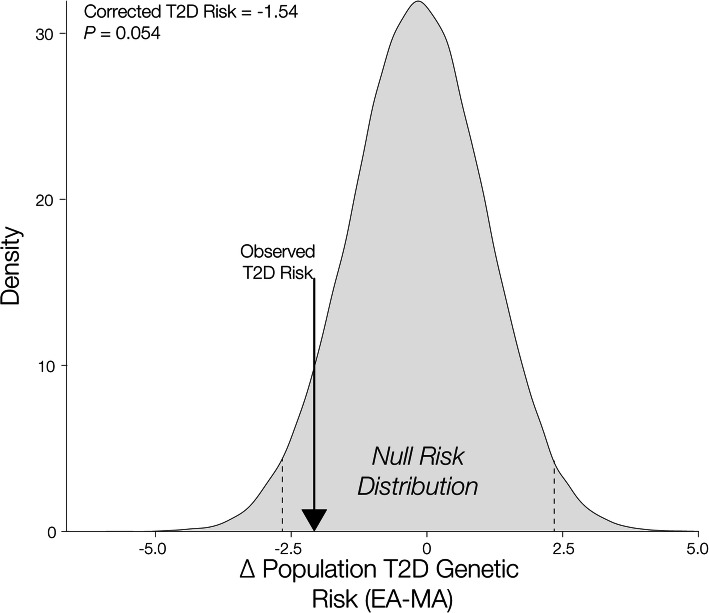
Correcting for ancestry bias in T2D risk inference. The null distribution of T2D relative risk between the EA-MA populations is generated by randomly sampling risk alleles with the same frequency of ancestral/derived alleles as the GWAS source population. The initial observed T2D relative risk is compared to the null distribution to yield a corrected score. The observed T2 relative risk is shown on the distribution (arrow) along with the corrected T2D relative risk value and its significance (upper left)

An example of this control can be seen for the comparison of T2D genetic risk between the EA and MA populations (Fig. 7). The observed value of *∆PRS* for EA-MA is − 2.08, while the null *∆PRS* distribution is centered around 0 with a mean value of − 0.16 and a standard deviation of 1.25. Thus, there is a slight bias in PRS calculation for the two populations. Accordingly, correcting for SNP ascertainment bias does mitigate the difference in predicted risk between the two populations, with a corrected *∆PRS* value of 1.54 that is marginally significant at *P* = 0.054. Given what we know about the higher prevalence of T2D in the MA population, we may consider this correction to be accurate, in the sense that it preserves the direction of the genetic risk difference, but conservative as it dampens the observed effect.

## Discussion

HL populations are burdened with a high and increasing prevalence of T2D, both in the US and in Latin America (Fig. 1) [5, 6]. Recent developments in the estimation of genetic risk using PRS provide opportunities to reduce this burden through improved screening and prevention efforts [10]. Nevertheless, there are a number of challenges that need to be met in order to ensure that genetic risk of T2D, and other common heritable diseases, can be accurately predicted using PRS [13, 14]. In particular, the bias towards European ancestry cohorts in GWAS [20, 21] has the potential to limit the utility of PRS in HL populations. In addition, the extremely diverse ancestries that can be found among HL populations could lead to mis-estimation of genetic risk for distinct HL subgroups.

There are two broad solutions to these ancestry-related challenges to genetic risk inference: (i) more data and (ii) better methods. Obviously, more GWAS that include cohorts that capture the genetic diversity of HL populations will go a long way towards providing the raw material, in the form of risk increasing genetic variants relevant to those same populations, which are needed to compute accurate PRS. However, given the current pace of efforts to diversify GWAS, along with the very high cost of these studies, it is unrealistic to expect the GWAS coverage of HL populations to approach that of European ancestry cohorts any time soon. In the meantime, new methods that explicitly leverage ancestry, e.g. modeling differences in allele frequencies across populations, may help to increase confidence in cross-population PRS calculation.

Here, we have shown that considering the consistency of GWAS variant effects across populations and modeling population-specific allele frequencies can increase confidence in cross-population PRS. T2D is a special case with respect to common heritable diseases in the sense that it has been extensively studied via numerous GWAS, and it has the most diverse set of ancestry cohorts seen for any GWAS trait (Fig. 2) [11]. In addition, recent studies have combined cohorts from different ancestries to increase confidence in the discovery of T2D associated variants [34, 35]. These facts allowed us to evaluate the extent to which GWAS variants discovered in cohorts with different ancestries yield similar PRS. The signal of T2D relative risk in Colombia is highly similar when GWAS variants discovered in different ancestry cohorts are used for PRS (Fig. 4). A similar result was seen for T2D risk in the US, but in this case, consistency of T2D associations across cohorts seemed to provide more reliable PRS estimates (Fig. 6). Finally, we proposed a conservative control for cross-population PRS inference based on modeling the frequencies of ancestral and derived alleles in the different populations being considered (Fig. 7).

A recent study compared the utility of GWAS SNPs ascertained from EA versus HL populations for a calculating PRS in HL populations across twelve different traits [53]. While there was a wide variety of relative performance of EA SNPs across the traits, the majority of EA SNP sets showed comparable risk prediction accuracy compared to the best performing SNP sets, which included information from HL GWAS cohorts. Nevertheless, the inclusion of non-EA GWAS association results to refine the SNP weights improved accuracy across the board. The results are consistent with our own findings suggesting that information from multi-ethnic GWAS cohorts can be used to refine PRS inference.

## Conclusions

One promising area for future work entails the application of machine learning methods to the inference of polygenic risk [54]. Currently, PRS calculations are based on GWAS that explicitly assume an additive model of genetic effects on traits of interest. Accordingly, standard methods for computing PRS, such as the kind we use here, entail a straightforward summation of risk alleles genome-wide. Of course, it may be more biologically realistic to assume that there are non-additive genetic effects among variants discovered by GWAS and used for PRS. If this is indeed the case, then more sophisticated machine learning algorithms may ultimately improve the accuracy of PRS calculation. The use of machine learning for polygenic risk inference is still in the very early stages; it remains to be seen if this approach will yield demonstrable improvement over current best practices.

The control we developed here for cross-population PRS inference is based on differences in ancestral versus derived allele frequencies among populations with distinct ancestry profiles. However, differences in LD across populations with divergent ancestries can also confound cross-population PRS inference. This is particularly true for African ancestry populations, which tend to have short and distinct LD blocks compared to non-African populations. Accordingly, controlling for such differences provides another promising approach for improving cross-population PRS inference. Indeed, a previous study has shown that accommodating differences in LD patterns across populations can substantially improve the accuracy of PRS computed for distinct ancestry cohorts [55]. In the future, we plan to combine allele frequency and LD based approaches to improving the accuracy cross-population PRS.

We employed a population-level approach to T2D genetic risk inference and evaluation in this study, comparing T2D relative genetic risk between populations to population-specific ancestry profiles and epidemiological data on observed T2D prevalence. Taken together with the robust collection of T2D variant associations from a number of diverse GWAS cohorts, this approach allowed us to broadly assess the impacts of ancestry on T2D genetic risk inference in HL populations. Going forward, a more rigorous assessment of PRS accuracy will require individual-level phenotype data, for both model training and test sets. Data of this kind are beginning to emerge thanks to the activity of a number of diabetes research consortia along with more broadly focused biobanks that collect patient genotypes and electronic health records. We anticipate that joint analysis of individual-level genotype-phenotype data gleaned from sources of this kind will help to further develop and validate ancestry-informed approaches to T2D genetic risk inference.

## Supplementary information

**Additional file 1: Table S1.** Summary statistics for type 2 diabetes (T2D) SNP associations from the EBI-NHGRI GWAS Catalog used for PRS calculations.

**Additional file 2.** Results on genetic ancestry and admixture for Colombian and US populations (Figure S1) and the effects of linkage disequilibrium (LD) on T2D genetic risk inference (Figures S2 and S3). Details on the control used to correct for ancestry bias in T2D risk inference.

## Data Availability

Whole genome genotype data for ChocoGen project donors - ~ 550 k SNPs for 98 individuals - are made available upon request (see https://www.chocogen.com/data). All other data used for the analysis are publicly available and can be accessed as described in the Methods section.

## Abbreviations

EA: European-American
GWAS: Genome-wide association study
HL: Hispanic/Latino
LD: Linkage disequilibrium
MA: Mexican-American
PRS: Polygenic risk score
T2D: Type 2 diabetes
WHO: World Health Organization

## References

[1] Zimmet PZ (2017). Diabetes and its drivers: the largest epidemic in human history?. Clin Diabetes Endocrinol.

[2] van Dieren S, Beulens JW, van der Schouw YT, Grobbee DE, Neal B (2010). The global burden of diabetes and its complications: an emerging pandemic. Eur J Cardiovasc Prev Rehabil.

[3] Herman WH, Zimmet P (2012). Type 2 diabetes: an epidemic requiring global attention and urgent action. Diabetes Care.

[4] IDF Diabetes Atlas, 8th Edition [http://www.diabetesatlas.org/] Accessed 3/6/2019.

[5] Spanakis EK, Golden SH (2013). Race/ethnic difference in diabetes and diabetic complications. Curr Diab Rep.

[6] Cusi K, Ocampo GL (2011). Unmet needs in Hispanic/Latino patients with type 2 diabetes mellitus. Am J Med.

[7] Meigs JB, Cupples LA, Wilson PW (2000). Parental transmission of type 2 diabetes: the Framingham offspring study. Diabetes.

[8] Poulsen P, Kyvik KO, Vaag A, Beck-Nielsen H (1999). Heritability of type II (non-insulin-dependent) diabetes mellitus and abnormal glucose tolerance – a population-based twin study. Diabetologia.

[9] Willemsen G, Ward KJ, Bell CG, Christensen K, Bowden J, Dalgard C, Harris JR, Kaprio J, Lyle R, Magnusson PK (2015). The concordance and heritability of type 2 diabetes in 34,166 twin pairs from international twin registers: the discordant twin (DISCOTWIN) consortium. Twin Res Hum Genet.

[10] Khera AV, Chaffin M, Aragam KG, Haas ME, Roselli C, Choi SH, Natarajan P, Lander ES, Lubitz SA, Ellinor PT (2018). Genome-wide polygenic scores for common diseases identify individuals with risk equivalent to monogenic mutations. Nat Genet.

[11] McMahon A, Malangone C, Suveges D, Sollis E, Cunningham F, Riat HS, MacArthur JAL, Hayhurst J, Morales J, Guillen JA (2018). The NHGRI-EBI GWAS catalog of published genome-wide association studies, targeted arrays and summary statistics 2019. Nucleic Acids Res.

[12] Chande AT, Norris ET, Rishishwar L, Jordan IK, Wang L, Conley AB, Valderrama-Aguirre A (2018). GlobAl distribution of GEnetic traits (GADGET) web server: polygenic trait scores worldwide. Nucleic Acids Res.

[13] Rosenberg NA, Edge MD, Pritchard JK, Feldman MW (2019). Interpreting polygenic scores, polygenic adaptation, and human phenotypic differences. Evol Med Public Health.

[14] De La Vega FM, Bustamante CD (2018). Polygenic risk scores: a biased prediction?. Genome Med.

[15] Marigorta UM, Navarro A (2013). High trans-ethnic replicability of GWAS results implies common causal variants. PLoS Genet.

[16] Marigorta UM, Rodríguez JA, Gibson G, Navarro A (2018). Replicability and prediction: lessons and challenges from GWAS. Trends Genet.

[17] Duncan L, Shen H, Gelaye B, Meijsen J, Ressler K, Feldman M, Peterson R, Domingue B. Analysis of polygenic risk score usage and performance in diverse human populations. Nat Commun. 2019;10(1):3328. 10.1038/s41467-019-11112-0 PMC6658471 31346163

[18] Kim MS, Patel KP, Teng AK, Berens AJ, Lachance J. Genetic disease risks can be misestimated across global populations. Genome Biol. 2018;19(1):179. 10.1186/s13059-018-1561-7 PMC6234640 30424772

[19] Martin AR, Gignoux CR, Walters RK, Wojcik GL, Neale BM, Gravel S, Daly MJ, Bustamante CD, Kenny EE (2017). Human demographic history impacts genetic risk prediction across diverse populations. Am J Hum Genet.

[20] Bustamante CD, Burchard EG, De la Vega FM (2011). Genomics for the world. Nature.

[21] Popejoy AB, Fullerton SM (2016). Genomics is failing on diversity. Nature.

[22] Mora GC (2014). Making Hispanics: how activists, bureaucrats, and media constructed a new American: University of Chicago Press.

[23] Ruiz-Linares A, Adhikari K, Acuna-Alonzo V, Quinto-Sanchez M, Jaramillo C, Arias W, Fuentes M, Pizarro M, Everardo P, de Avila F (2014). Admixture in Latin America: geographic structure, phenotypic diversity and self-perception of ancestry based on 7,342 individuals. PLoS Genet.

[24] Moreno-Estrada A, Gravel S, Zakharia F, McCauley JL, Byrnes JK, Gignoux CR, Ortiz-Tello PA, Martinez RJ, Hedges DJ, Morris RW (2013). Reconstructing the population genetic history of the Caribbean. PLoS Genet.

[25] Homburger JR, Moreno-Estrada A, Gignoux CR, Nelson D, Sanchez E, Ortiz-Tello P, Pons-Estel BA, Acevedo-Vasquez E, Miranda P, Langefeld CD (2015). Genomic insights into the ancestry and demographic history of South America. PLoS Genet.

[26] Wang S, Ray N, Rojas W, Parra MV, Bedoya G, Gallo C, Poletti G, Mazzotti G, Hill K, Hurtado AM (2008). Geographic patterns of genome admixture in Latin American mestizos. PLoS Genet.

[27] Bryc K, Velez C, Karafet T, Moreno-Estrada A, Reynolds A, Auton A, Hammer M, Bustamante CD, Ostrer H (2010). Colloquium paper: genome-wide patterns of population structure and admixture among Hispanic/Latino populations. Proc Natl Acad Sci U S A.

[28] Conley AB, Rishishwar L, Norris ET, Valderrama-Aguirre A, Mariño-Ramírez L, Medina-Rivas MA, Jordan IK (2017). A Comparative Analysis of Genetic Ancestry and Admixture in the Colombian Populations of Chocó and Medellín. G3 (Bethesda, Md).

[29] The World Bank Diabetes Prevalence [https://data.worldbank.org/indicator/SH.STA.DIAB.ZS] Accessed 12/17/2018.

[30] Statistics About Diabetes [http://www.diabetes.org/diabetes-basics/statistics/] Accessed 12/17/2018.

[31] Complete Health Indicator Report of Diabetes Prevalence [https://ibis.health.utah.gov/indicator/complete_profile/DiabPrev.html ] Accessed 12/6/2018.

[32] Health CoLAP (2012). Trends in Diabetes: Time for Action.

[33] Morales J, Welter D, Bowler EH, Cerezo M, Harris LW, McMahon AC, Hall P, Junkins HA, Milano A, Hastings E (2018). A standardized framework for representation of ancestry data in genomics studies, with application to the NHGRI-EBI GWAS catalog. Genome Biol.

[34] Morris AP, Voight BF, Teslovich TM, Ferreira T, Segrè AV, Steinthorsdottir V, Strawbridge RJ, Khan H, Grallert H, Mahajan A (2012). Large-scale association analysis provides insights into the genetic architecture and pathophysiology of type 2 diabetes. Nat Genet.

[35] Cho YS, Chen C-H, Hu C, Long J, Hee Ong RT, Sim X, Takeuchi F, Wu Y, Go MJ, Yamauchi T (2011). Meta-analysis of genome-wide association studies identifies eight new loci for type 2 diabetes in east Asians. Nat Genet.

[36] Auton A, Abecasis GR, Altshuler DM, Durbin RM, Abecasis GR, Bentley DR, Chakravarti A, Clark AG, Donnelly P, The Genomes Project C (2015). A global reference for human genetic variation. Nature.

[37] Medina-Rivas MA, Norris ET, Rishishwar L, Conley AB, Medrano-Trochez C, Valderrama-Aguirre A, Vannberg FO, Mariño-Ramírez L, Jordan IK (2016). Chocó, Colombia: a hotspot of human biodiversity. Revista biodiversidad neotropical.

[38] Delaneau O, Howie B, Cox AJ, Zagury J-F, Marchini J (2013). Haplotype estimation using sequencing reads. Am J Hum Genet.

[39] Delaneau O, Marchini J, Genomes Project C, Genomes Project C (2014). Integrating sequence and array data to create an improved 1000 Genomes Project haplotype reference panel. Nat Commun.

[40] Chande AT, Rowell J, Rishishwar L, Conley AB, Norris ET, Valderrama-Aguirre A, Medina-Rivas MA, Jordan IK (2017). Influence of genetic ancestry and socioeconomic status on type 2 diabetes in the diverse Colombian populations of Chocó and Antioquia. Sci Rep.

[41] Chang CC, Chow CC, Tellier LC, Vattikuti S, Purcell SM, Lee JJ (2015). Second-generation PLINK: rising to the challenge of larger and richer datasets. GigaScience.

[42] Vilhjálmsson Bjarni J, Yang J, Finucane Hilary K, Gusev A, Lindström S, Ripke S, Genovese G, Loh P-R, Bhatia G, Do R (2015). Modeling linkage disequilibrium increases accuracy of polygenic risk scores. Am J Hum Genet.

[43] Alexander DH, Novembre J, Lange K (2009). Fast model-based estimation of ancestry in unrelated individuals. Genome Res.

[44] Brancati FL, Kao WHL, Folsom AR, Watson RL, Szklo M (2000). Incident type 2 diabetes mellitus in African American and white AdultsThe atherosclerosis risk in communities study. JAMA.

[45] Burrows NR, Geiss LS, Engelgau MM, Acton KJ (2000). Prevalence of diabetes among native Americans and Alaska natives, 1990-1997: an increasing burden. Diabetes Care.

[46] Cowie CC, Rust KF, Byrd-Holt DD, Eberhardt MS, Flegal KM, Engelgau MM, Saydah SH, Williams DE, Geiss LS, Gregg EW (2006). Prevalence of diabetes and impaired fasting glucose in adults in the U.S. population: National Health and Nutrition Examination Survey 1999-2002. Diabetes Care.

[47] Cowie CC, Rust KF, Ford ES, Eberhardt MS, Byrd-Holt DD, Li C, Williams DE, Gregg EW, Bainbridge KE, Saydah SH (2009). Full accounting of diabetes and pre-diabetes in the U.S. population in 1988-1994 and 2005-2006. Diabetes Care.

[48] Maskarinec G, Grandinetti A, Matsuura G, Sharma S, Mau M, Henderson BE, Kolonel LN (2009). Diabetes prevalence and body mass index differ by ethnicity: the multiethnic cohort. Ethnicity & disease.

[49] Chacon-Duque JC, Adhikari K, Fuentes-Guajardo M, Mendoza-Revilla J, Acuna-Alonzo V, Barquera R, Quinto-Sanchez M, Gomez-Valdes J, Everardo Martinez P, Villamil-Ramirez H (2018). Latin Americans show wide-spread Converso ancestry and imprint of local native ancestry on physical appearance. Nat Commun.

[50] Moreno-Estrada A, Gignoux CR, Fernandez-Lopez JC, Zakharia F, Sikora M, Contreras AV, Acuna-Alonzo V, Sandoval K, Eng C, Romero-Hidalgo S (2014). Human genetics. The genetics of Mexico recapitulates native American substructure and affects biomedical traits. Science.

[51] Cheng CY, Reich D, Haiman CA, Tandon A, Patterson N, Selvin E, Akylbekova EL, Brancati FL, Coresh J, Boerwinkle E (2012). African ancestry and its correlation to type 2 diabetes in African Americans: a genetic admixture analysis in three U.S. population cohorts. PLoS One.

[52] Lachance J, Tishkoff SA (2013). SNP ascertainment bias in population genetic analyses: why it is important, and how to correct it. Bioessays.

[53] Grinde KE, Qi Q, Thornton TA, Liu S, Shadyab AH, Chan KHK, Reiner AP, Sofer T (2019). Generalizing polygenic risk scores from Europeans to Hispanics/Latinos. Genet Epidemiol.

[54] Ho DSW, Schierding W, Wake M, Saffery R, O'Sullivan J (2019). Machine learning SNP based prediction for precision medicine. Front Genet.

[55] Márquez-Luna C, Loh P-R, Price AL (2017). Multiethnic polygenic risk scores improve risk prediction in diverse populations. Genet Epidemiol.

