# Supplementary material for: Ancestry effects on type 2 diabetes genetic risk inference in Hispanic/Latino populations
Source: BMC Med Genet. 2020 Jun 25;21(Suppl 2):132. doi: 10.1186/s12881-020-01068-0 (PMC7315475; doi:10.1186/s12881-020-01068-0)
Supplement: Supplementary file 2 — Additional file 2. Results on genetic ancestry and admixture for Colombian and US populations (Figure S1) and the effects of linkage disequilibrium (LD) on T2D genetic risk inference (Figures S2 and S3). Details on the control used to correct for ancestry bias in T2D risk inference. [file 12881_2020_1068_MOESM2_ESM.pdf]

Supplementary Material for:

Ancestry effects on type 2 diabetes genetic risk inference in Hispanic/Latino populations

Aroon T. Chande, Lavanya Rishishwar, Andrew B. Conley, Augusto Valderrama-Aguirre, Miguel A. Medina-Rivas, and I. King Jordan

Contents

Genetic ancestry and admixture for Colombian and US populations 2

Effects of linkage disequilibrium (LD) on T2D genetic risk inference 3

Correcting for ancestry bias in T2D risk inference 5

References 8

Genetic ancestry and admixture for Colombian and US populations

Whole genome genotypes from the HL populations in Colombia and the US were compared to genotype data from proxy source populations in Africa, Europe, and the Americas (Table 1), using the program ADMIXTURE [1], in order to calculate three-way ancestry percentages for each individual genome (Supplementary Figure S1A). The resulting ancestry percentages were then regressed against predicted T2D PRS for these same individuals as shown in Figures 3B and 5B. Details of this analysis can be found in the Methods section. We also performed local ancestry analysis, i.e. ancestry assignment for specific haplotypes genome-wide, using the program RFMix [2]. The local ancestry estimates from RFMix were summed across the entire genomes to yield global ancestry estimates and these were compared to the estimates from ADMXITURE to further verify ancestry percentages (Supplementary Figure S1B).

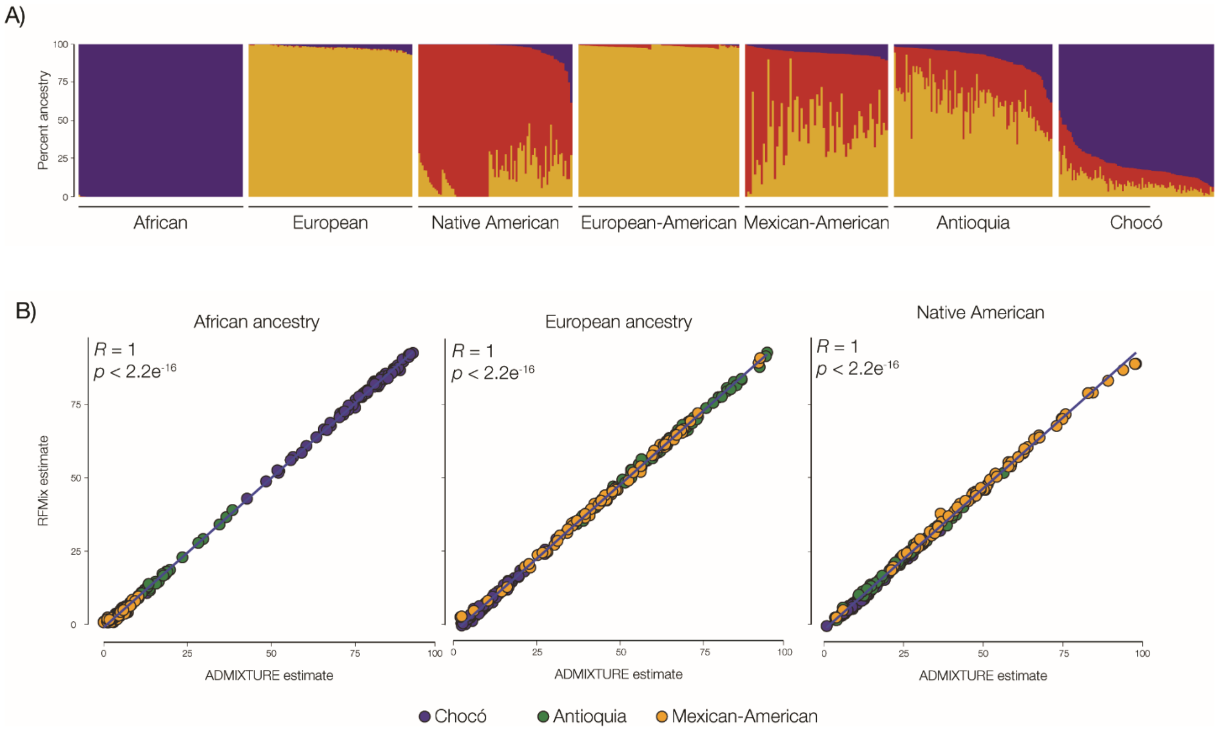

Figure S1. **Ancestry and admixture patterns for the Colombian and US populations studied here.** (A) Ancestry percentages – African (blue), European (yellow), and Native American (red) – are shown for individuals sampled from proxy ancestral source populations in Africa, Europe and the Americas along with the admixed Colombian (Antioquia and Chocó) and US populations (European- and Mexican-American). Each stacked bar represents the relative ancestry percentages for one individual from a given population. The population sources for this analysis are shown in Table 1. (B) Comparison between ADMIXTURE (x-axis) and RFMix (y-axis) continental ancestry percent estimates for the admixed populations analyzed here. Individuals form each admixed population are color coded as shown.

Effects of linkage disequilibrium (LD) on T2D genetic risk inference

Divergent patterns of LD across populations with distinct ancestry can confound cross-population PRS inference. We first controlled for the effects of LD by performing LD pruning on the initial set of 165 T2D-associated SNPs used to compute PRS in the Colombian and US populations. LD pruning was performed for all four populations together by removing variants that are linked at $r^2 > 0.1$, retaining the linked SNP with the highest minor allele frequency. Details of this analysis can be found in the Methods section. Our approach to LD pruning was intended to be very conservative (stringent) in terms of both the low r^2 threshold and the combined use of the four populations. Consistent with this intention, LD pruning reduced the total number of T2D associated SNPs for PRS analysis from 165 to 42. Nevertheless, the signal of relative T2D risk between populations remains the same for both Colombia and the US (Figure S2).

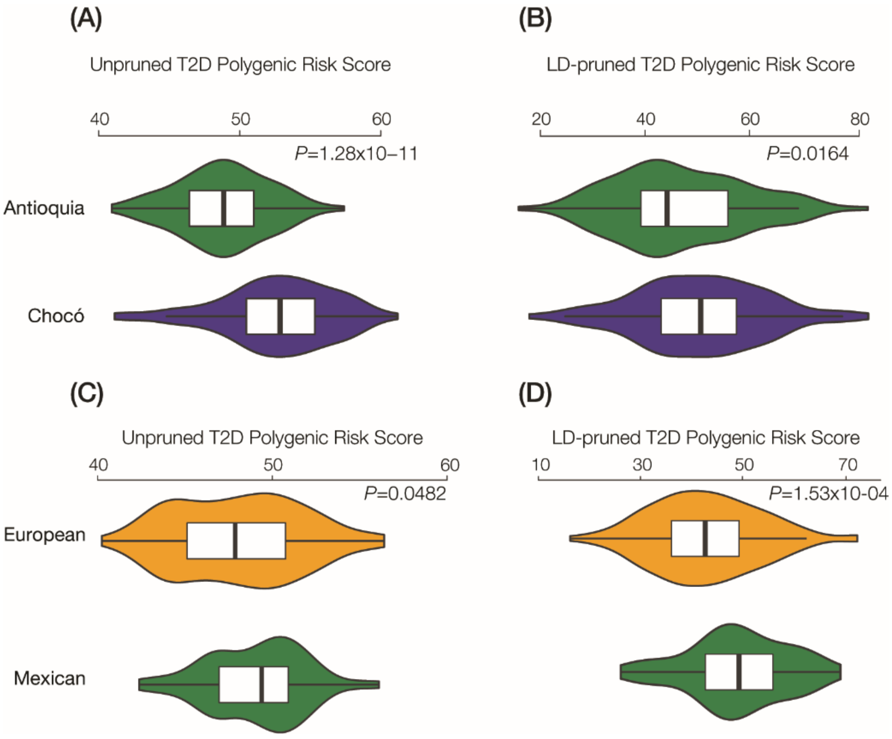

Figure S2. **Effects of linkage disequilibrium (LD) pruning on T2D genetic risk inference.** Polygenic risk scores were calculated and presented as shown in Figures 3 and 5, using both the full unpruned set of T2D associated SNPs (*n*=165) and the reduced LD pruned set (*n*=42). Results are shown for the Colombian (panels A and B) and US populations (panels C and D).

We further attempted to control for LD differences in cross-population PRS by using the LDpred program to compute PRS for the EA and MA populations using the DIAGRAM multi-ethnic GWAS T2D SNP-association data. LDpred performs LD clumping, to correct for LD structure, along with re-weighting on SNP effect sizes, by choosing the most informative SNP within any given LD window [3]. We ran LDpred across of series of *P*-value thresholds (Figure S3 panels B-E), and found the results to be almost entirely consistent with the original unpruned set (Figure S3 panel A).

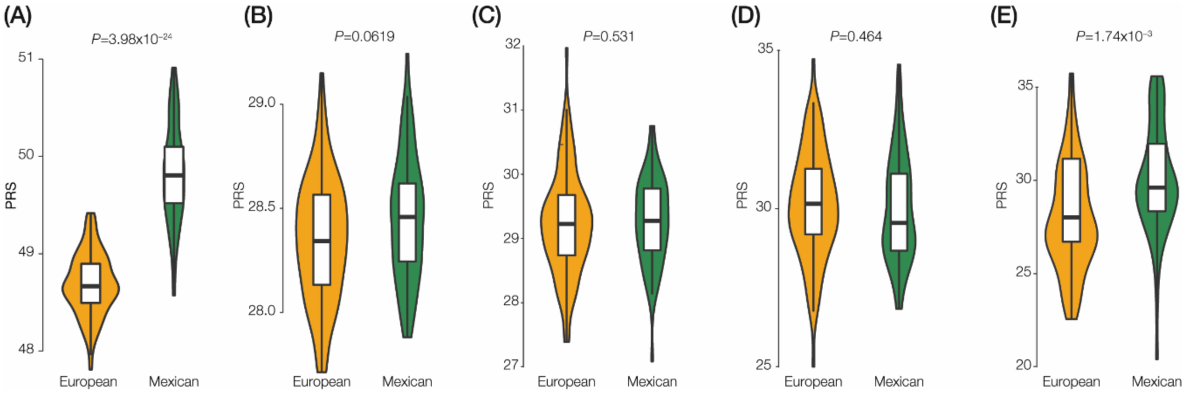

Figure S3. **Effects of linkage disequilibrium (LD) clumping and *P*-value thresholding on T2D polygenic risk scores**. Distributions of population PRS using all marginally-significant variant effects from GWAS summary data (A) and from LD clumping and *P*-value thresholding using an LD cutoff of $r^2 > 0.1$ and *P*-value cutoffs of $1x10^{-1}$ (B), $1x10^{-2}$ (C), $1x10^{-3}$ (D), and $1x10^{-4}$ (E).

There are other programs available for controlling for LD when computing PRS – lassosum and PRSice-2 – but these programs use individual level phenotype data to optimize the selection of SNPs to be included in PRS tests [4, 5]. Lassosum can be run in the pseudo validation mode without phenotypes, but doing so here on the DIAGRAM data simply yields overlapping PRS distributions between populations. Running PRSice-2 without phenotype data yields an error message and does not produce any output.

Correcting for ancestry bias in T2D risk inference

Our control for ancestry bias in polygenic risk inference is presented in the main text; additional details of this process are given below.

1. Collect trait SNP set and calculate population-specific PRS values and between-population PRS differences (ΔPRS).

   1.1. SNP sets for traits of interest can be curated from the NHGRI-EBI GWAS Catalog at https://www.ebi.ac.uk/gwas/ or from GWAS consortium resource webpages, such as the DIAGRAM page used to curate T2D SNPs here http://diagram-consortium.org/downloads.html. SNP sets can also be curated from the literature using the NCBI PubMed database https://www.ncbi.nlm.nih.gov/pubmed/. In the case of literature searches for trait SNP sets, we recommend using the most up-to-date and comprehensive meta-analyses that can be found for any trait of interest.

   1.2. PRS can be computed from whole genome genotype data by evaluating the numbers of risk alleles present in any individual genome (genotype) being analyzed. Unweighted PRS can be computed as:

$$PRS = \sum\nolimits_{i=1}^{n} G_i \Big/ \sum\nolimits_{i=1}^{n} A_i$$

   where $G_i \in \{0,1,2\}$ corresponds to homozygous absent, heterozygous, and homozygous present effect alleles for each T2D SNP *i* and $A_i \in \{0,1,2\}$ corresponding the total number of alleles with variant calls at each SNP *i*. Weighted PRS can be computed as:

$$PRS = \sum\nolimits_{i=1}^{n} G_i \times e_i \Big/ \sum\nolimits_{i=1}^{n} A_i$$

   where e_i corresponds to the allele-specific effect size. Note that unweighted PRS should be used when trait SNPs are curated from multiple studies, owing to the fact that effect sizes from different studies cannot be accurately combined.

1.3. Between-population PRS differences (ΔPRS) are computed as the difference between mean population-specific PRS values. For example, for the EA and MA populations, ΔPRS is computed as

$$\Delta PRS = \frac{\sum_{i=1}^{n} PRS_{EA}}{n_{EA}} - \frac{\sum_{i=1}^{n} PRS_{MA}}{n_{MA}}$$

2. Determine the distribution of derived allele frequencies (DAF) for trait-associated SNPs in the GWAS cohort source population.

   2.1. PRS are computed with biallelic SNPs taken from the 1000 Genomes Project phase 3 release VCF file. This file designates each allele as ancestral or derived in the 'AA' field of the 'INFO' column.

   2.2. The derived allele frequencies for (DAF) for trait-associated SNPs in the GWAS catalog are then calculated as shown in lines 1-6 of the pseudo-code (Figure S4).

3. Randomly sample SNP sets parameterized by this DAF distribution based on the DAFs from the distinct populations being compared (thereby eliminating between-population DAF biases).

   3.1. The derived allele frequency (DAF) in the GWAS catalog is represented as D as shown in line 6 of the pseudo-code, and the binomial probability of sampling an ancestral or derived allele is used to randomly select SNPs as shown in lines 7-11 of the pseudo-code.

4. Calculate between-population ΔPRS for all randomly sampled SNP sets and determine the null ΔPRS distribution.

   4.1. For each simulation, population-specific PRS values for the randomly simulated SNP sets are computed as shown in #1.2 above, and the mean population-specific PRS values are then used to compute the ΔPRS value as shown in #1.3 above.

5. Compare the observed ΔPRS to the null ΔPRS distribution and compute a z-score as the ancestry-corrected ΔPRS.

   5.1. The procedure in #4 above yields a null distribution of ΔPRS values.

5.2. The null ΔPRS value distribution from the simulated data is compared against the observed between population ΔPRS value to derive the ancestry corrected ΔPRS: $corr.\Delta PRS = (obs\Delta PRS - \mu_{null\Delta PRS})/\sigma_{null\Delta PRS}$.

```
For every SNP-Trait association in GWAS catalog
    For j = MA, EA
        Is the trait risk allele Ancestral or Derived?
            Increment Ancestral or Derived count as appropriate
            Save SNP-Trait association with Ancestral information
D = Compute DAF of GWAS SNP-Trait association
For i = 1 to 500,000
    For j = MA, EA
        For k = 1 to size of trait to simulate
            Decide whether to pick an Ancestral or Derived SNP-
            Trait associations using Binomial([Ancestral, Derived],
            D)
                Randomly select SNP-Trait association
        Compute PRS
    Compute ΔPRS
Compute mean and standard deviation of simulated ΔPRS distribution
```

Figure S4. **Pseudo-code for the derived allele frequency (DAF) parameterized simulation.** Individual steps in the control for PRS ancestry bias are enumerated above, with the correspondence of each step to the pseudo-code indicated.

References

1. Alexander DH, Novembre J, Lange K: **Fast model-based estimation of ancestry in unrelated individuals**. *Genome research* 2009, **19**(9):1655-1664.
2. Maples BK, Gravel S, Kenny EE, Bustamante CD: **RFMix: a discriminative modeling approach for rapid and robust local-ancestry inference**. *Am J Hum Genet* 2013, **93**(2):278-288.
3. Vilhjálmsson Bjarni J, Yang J, Finucane Hilary K, Gusev A, Lindström S, Ripke S, Genovese G, Loh P-R, Bhatia G, Do R *et al*: **Modeling Linkage Disequilibrium Increases Accuracy of Polygenic Risk Scores**. *The American Journal of Human Genetics* 2015, **97**(4):576-592.
4. Mak TSH, Porsch RM, Choi SW, Zhou X, Sham PC: **Polygenic scores via penalized regression on summary statistics**. *Genet Epidemiol* 2017, **41**(6):469-480.
5. Euesden J, Lewis CM, O'Reilly PF: **PRSice: Polygenic Risk Score software**. *Bioinformatics* 2015, **31**(9):1466-1468.
